# Anilinoquinoline based inhibitors of trypanosomatid proliferation

**DOI:** 10.1371/journal.pntd.0006834

**Published:** 2018-11-26

**Authors:** Lori Ferrins, Amrita Sharma, Sarah M. Thomas, Naimee Mehta, Jessey Erath, Scott Tanghe, Susan E. Leed, Ana Rodriguez, Kojo Mensa-Wilmot, Richard J. Sciotti, Kirsten Gillingwater, Michael P. Pollastri

**Affiliations:** 1 Northeastern University, Department of Chemistry & Chemical Biology, Boston, United States of America; 2 University of Georgia, Department of Cellular Biology, Athens, United States of America; 3 New York University School of Medicine, Department of Microbiology, New York, United States of America; 4 Anti-Infectives Screening Core, New York University School of Medicine, New York; 5 Experimental Therapeutics, Walter Reed Army Institute for Research, Silver Spring, United States of America; 6 Swiss Tropical and Public Health Institute, Department of Medical Parasitology and Infection Biology, Socinstrasse 57, Basel, Switzerland; 7 University of Basel, Petersplatz 1, Basel, Switzerland; McGill University, CANADA

## Abstract

We recently reported the medicinal chemistry re-optimization of a series of compounds derived from the human tyrosine kinase inhibitor, lapatinib, for activity against *Plasmodium falciparum*. From this same library of compounds, we now report potent compounds against *Trypanosoma brucei brucei* (which causes human African trypanosomiasis), *T*. *cruzi* (the pathogen that causes Chagas disease), and *Leishmania* spp. (which cause leishmaniasis). In addition, sub-micromolar compounds were identified that inhibit proliferation of the parasites that cause African animal trypanosomiasis, *T*. *congolense* and *T*. *vivax*. We have found that this set of compounds display acceptable physicochemical properties and represent progress towards identification of lead compounds to combat several neglected tropical diseases.

## Author summary

As part of our efforts to identify compounds that are active against the parasite that causes malaria (*P*. *falciparum*), we employed a “parasite hopping” approach in our drug discovery efforts. This involved screening a library of demonstrated antiparasitic agents against other parasites responsible for a host of neglected tropical diseases (NTDs) including Chagas disease (*T*. *cruzi*), human African trypanosomiasis (*T*. *brucei*) and cutaneous leishmaniasis (*L*. *major*). The compounds we identified generally show improved selectivity for the parasite of interest over the mammalian cell lines tested and, from this work, we have made progress towards the identification of lead compounds against multiple NTDs.

## Introduction

Neglected tropical diseases (NTDs) are a collection of 20 communicable diseases [1]. Though mostly treatable and/or preventable, NTDs remain a leading cause of morbidity and mortality affecting over 1 billion people worldwide. Many of the current treatments have significant side effects, poor efficacy and there are increasing reports of resistance in the literature [2, 3] highlighting the need for novel chemotherapeutics. Collectively, 17 of these NTDs account for 26 million disability-adjusted life years (DALY) [4], which is a sum of years of life lost due to premature mortality and those lost due to ill health or disability. In addition, livestock are also susceptible to various parasitic infections, and can lead to the development of diseases such as African animal trypanosomiasis (AAT), which has devastating economic effects on communities. AAT is caused by infection with *T*. *congolense* or *T*. *vivax* [5], and of the three main chemotherapeutics currently listed for treatment, diminazene aceturate and isometamidium chloride are the most widely used [6], though reports of resistance are increasing to both of these [7–8].

**Fig 1:**
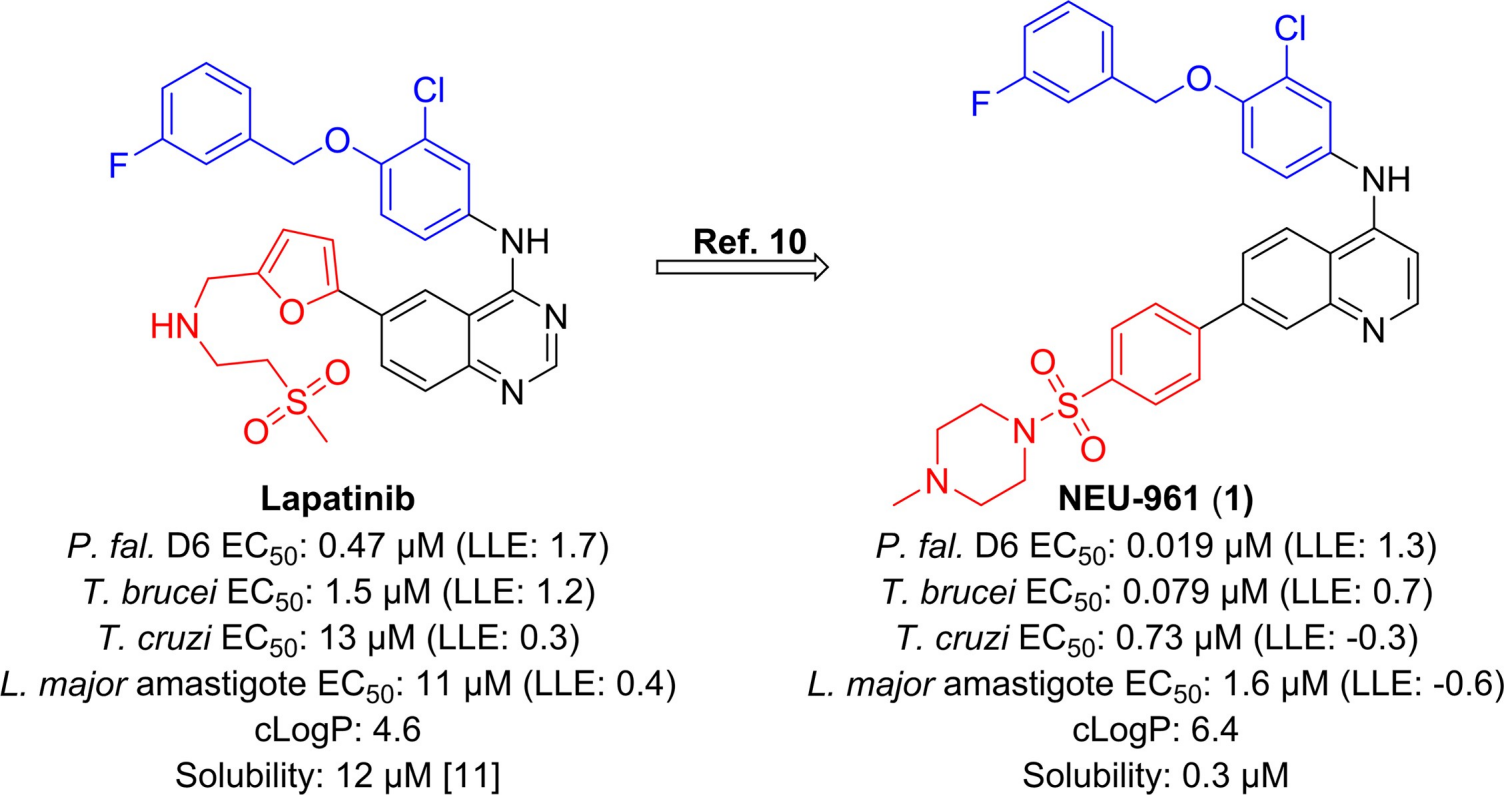
Project progression has led to the identification of potent inhibitors of *P*. *falciparum*, *T*.*b*. *brucei*, *T*. *cruzi*, and *L*. *major* through variations of the head region (shown in blue) and the linker/tail region (shown in red) from lapatinib [12] to compounds typified by 1. Desired ranges for physicochemical properties: cLogP: ≤3, lipophilic ligand efficiency (LLE = pEC_50_—cLogP): ≥4 [13], aqueous solubility: > 10 μM.

As part of our efforts to identify new drugs for human African trypanosomiasis (HAT), we undertook a target class repurposing project [9], wherein we screened known human tyrosine kinase inhibitors against a number of pathogenic protozoan parasites (**Fig 1**). Historically, we have cross-screened our compounds against *P*. *falciparum*, *T*.*b*. *brucei*, *T*. *cruzi*, and *L*. *major* to fully leverage chemical space covered by our optimization campaign [10]. This cross-screening led to the identification of **1** [10] as a potent inhibitor of proliferation of *P*. *falciparum*, *T*.*b*. *brucei* and *T*. *cruzi*. Herein, we report the results of a screening campaign using a set of previously reported, structurally related compounds previously optimized for their activity against *P*. *falciparum* [11]. We screened these compounds for activity against *T*.*b*. *brucei*, *T*. *cruzi* and *L*. *major* and we also report activity data for a subset of these compounds against *T*. *congolense* and *T*. *vivax*. The previously reported *P*. *falciparum* data for these compounds, along with additional screening data that is not discussed directly in this study, is provided in the **Supplementary Information, S1 Table.**

Established target product profiles for Chagas disease, leishmaniasis and HAT, were used to define hit and lead candidate criteria (summarized in the **Supplementary Information, S2 Table**) [14–15]. We note that, according to a committee coordinated by the Global Health Innovative Technology Fund (GHIT), a hit compound for Chagas disease or leishmaniasis should have an EC_50_ < 10 μM [15].

## Results and discussion

### Trypanosoma cruzi

**Fig 2:**
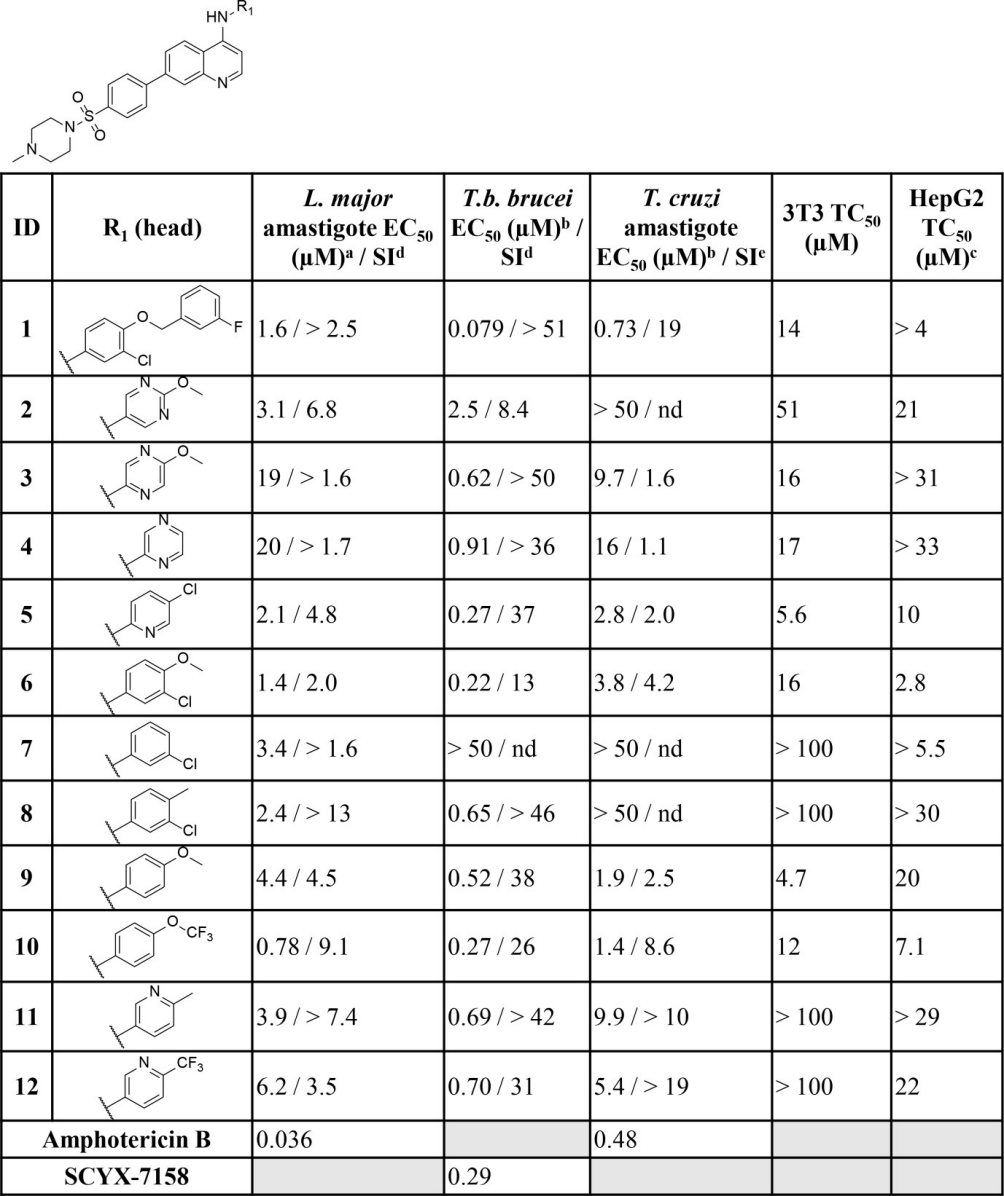
Biological activity of key compounds with varying head groups (denoted by R^1^) against *L*. *major* amastigote, *T*.*b*. *brucei*, *T*. *cruzi* cultures and HepG2 human cells. Data for the full screening set is summarized in the supplementary information. nd = not determined ^a^All r^2^ values are >0.9 unless noted otherwise ^b^All SEM values within 25% except for **1** where EC_50_ *T*. *cruzi* 0.73 (SEM: 0.2; 27%) ^c^Tested concentration ranges were determined based on compound solubility ^d^Selectivity index (SI) calculated relative to HepG2 cells. ^e^Selectivity index (SI) calculated relative to 3T3 cells.

**Fig 3:**
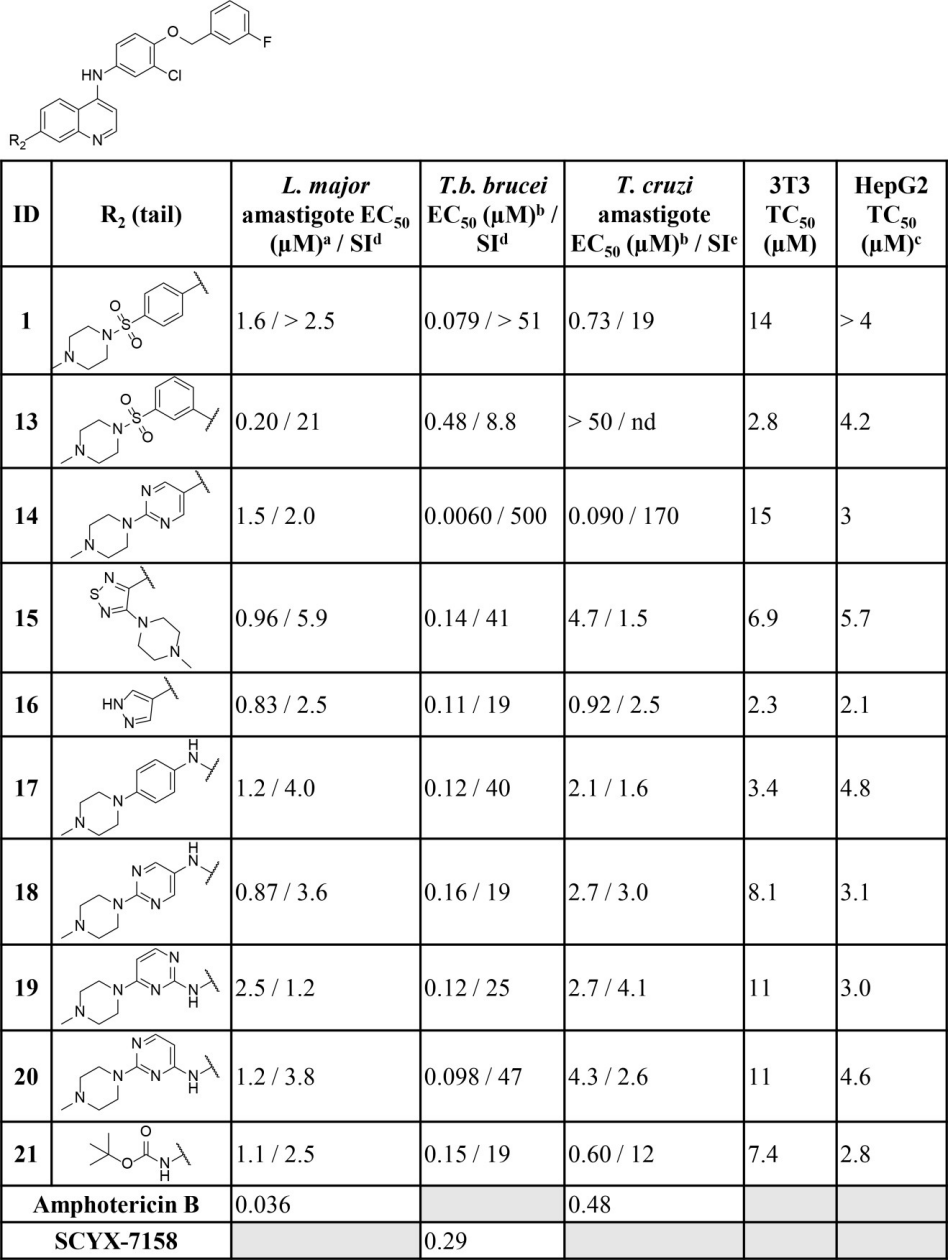
Biological activity of key compounds with varying tail groups (denoted by R^2^) against *L*. *major* amastigote, *T*.*b*. *brucei*, *T*. *cruzi* cultures and HepG2 human cells. Data for the full screening set is summarized in the supplementary information. nd = not determined ^a^All r^2^ values are >0.9 unless noted otherwise ^b^All SEM values within 25% except for **1** where EC_50_ *T*. *cruzi* 0.73 (SEM: 0.2; 27%) ^c^Tested concentration ranges were determined based on compound solubility ^d^Selectivity index (SI) calculated relative to HepG2 cells. ^e^Selectivity index (SI) calculated relative to 3T3 cells.

**Fig 4:**
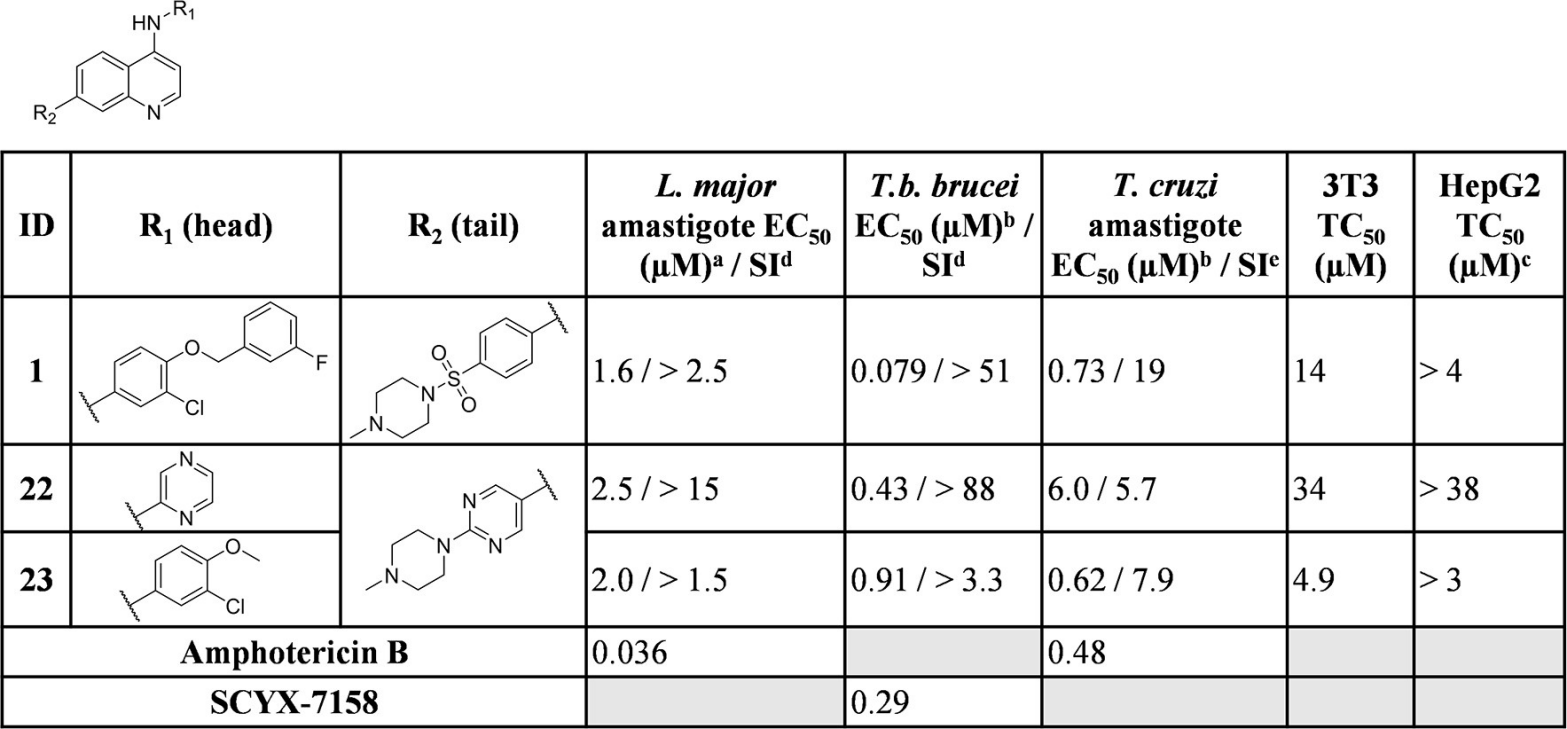
Biological activity of key compounds that combine head (R^1^) and tail (R^2^) groups against *L*. *major* amastigote, *T*.*b*. *brucei*, *T*. *cruzi* cultures and HepG2 human cells. Data for the full screening set is summarized in the supplementary information. nd = not determined ^a^All r^2^ values are >0.9 unless noted otherwise ^b^All SEM values within 25% except for **1** where EC_50_ *T*. *cruzi* 0.73 (SEM: 0.2; 27%) ^c^Tested concentration ranges were determined based on compound solubility ^d^Selectivity index (SI) calculated relative to HepG2 cells. ^e^Selectivity index (SI) calculated relative to 3T3 cells.

**Fig 5:**
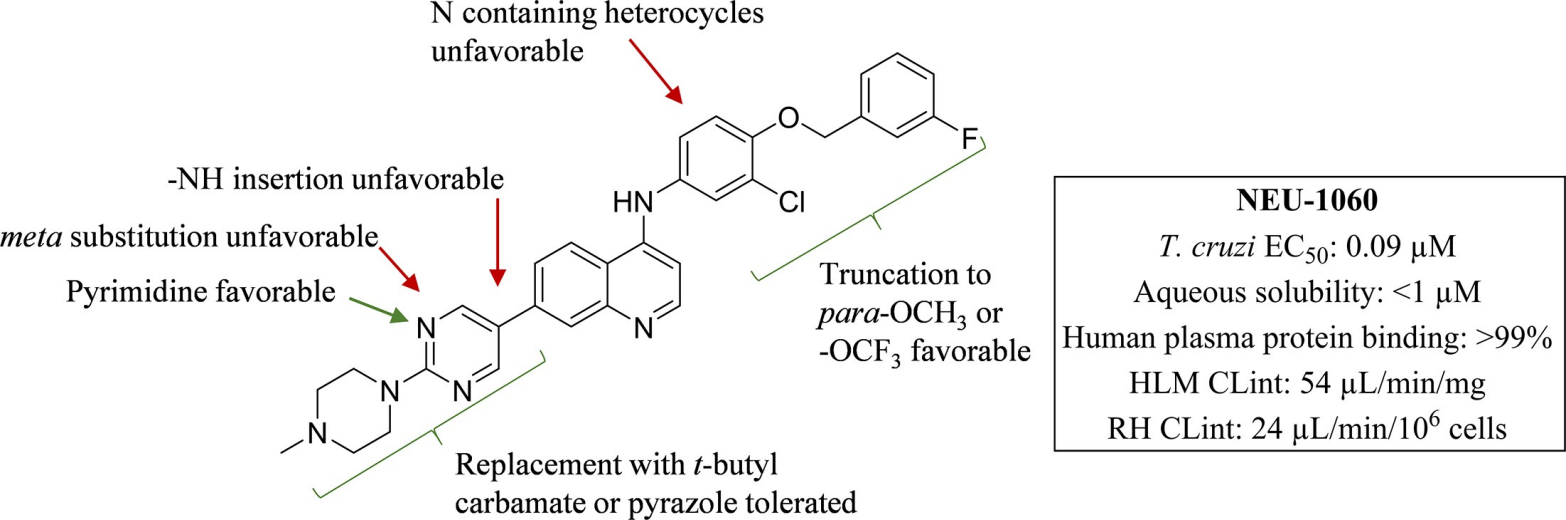
SAR summary around this series for potent anti-*T*. *cruzi* activity.

When tested against intracellular *T*. *cruzi* parasites, several compounds displayed low micromolar inhibition of *T*. *cruzi*, and these included truncated head groups 3-chloro-4-methoxyphenyl (**6**, EC_50_: 3.8 μM) and *para*-methoxyphenyl (**9**, EC_50_: 1.9 μM), as well as the *para*-trifluoromethoxyphenyl (**10**, EC_50_: 1.4 μM) analog (**Fig 2**). Removal of the phenylsulfonamide and replacement with the *para-*substituted pyrimidine (**14**, EC_50_: 0.090 μM) (**Fig 3**) led to the most potent compound against *T*. *cruzi* identified in this series and afforded a significant boost in selectivity index (SI) versus 3T3 cells (SI: 170). Other submicromolar compounds were identified, such as the pyrazole analog (**16**, EC_50_: 0.92 μM) and the *t*-butyl carbamate (**21**, EC_50_: 0.60 μM), though selectivity versus 3T3 cells was an issue for **16** in particular (SI: 2.5). An additional compound, **23** (EC_50_: 0.62 μM) (**Fig 4**), had an improved lipophilic ligand efficiency [13] (LLE; 1.8 compared to -0.29 for **1**, see **Supplementary Information, S3 Table** for a complete list of values) and maintained potent inhibition of *T*. *cruzi*, though the aqueous solubility was poor (<1 μM, see **Supplementary Information, S4 Table** for complete list of ADME values) and the human liver microsome intrinsic clearance (HLM Cl_int_: 170 μL/min/mg), and rat hepatocyte intrinsic clearance (RH Cl_int_: 45 μL/min/10^6^ cells) were both high. Importantly, host cell toxicity (3T3 cells) was generally low for the series, and the selectivity index (SI) was above the targeted 10×EC_50_ threshold. The general SAR trends observed for this series against *T*. *cruzi* are summarized in **Fig 5**.

### Leishmania major

**Fig 6:**
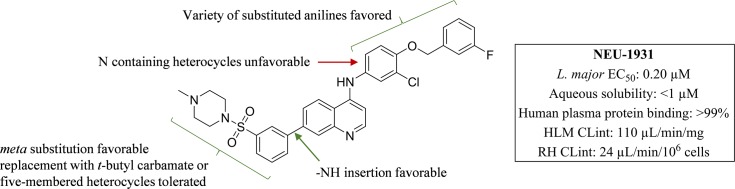
Series SAR summary for anti*-L*. *major* activity.

Some of the SAR trends observed with *T*. *cruzi* were mirrored in *L*. *major*. Truncation of the head group to the 3-chloro-4-methoxyphenyl (**6**, EC_50_: 1.4 μM), 3-chlorophenyl (**7**, EC_50_: 3.4 μM), *para*-methoxyphenyl (**9**, EC_50_: 4.4 μM), and the *para-*trifluoromethoxyphenyl (**10**, EC_50_: 0.78 μM) all led to potent analogs (**Fig 2**). In addition, several pyridyl (**5**, and **11**), and pyrimidine (**2**) derivatives exhibited potent inhibition (EC_50_ values ranging from 2.1–3.9 μM). The *meta*- substituted sulfonamide analog (**13**) was the most potent compound identified (EC_50_: 0.20 μM) and suggested that the change in vector may be favorable (**Fig 3**). This idea was supported by the thiadiazole analog **15** (EC_50_: 0.96 μM) which possesses a more acute vector than **13** yet maintains potency. In addition, compound activity remained upon removal of the tail group (red in **Fig 1**) and replacement with the *t*-butyl carbamate (**21**, EC_50_: 1.1 μM) or pyrazole (**16**, EC_50_: 0.83 μM). Finally, removal of the sulfonamide and replacement with the pyrimidine (**14**; EC_50_: 1.5 μM), as well as incorporation of an -NH linker (**17**–**20**), led to several compounds with low-micromolar inhibition (EC_50_ values ranging from 0.87–2.5 μM) of *L*. *major*. Importantly, host cell toxicity (HepG2) was found to be low for these analogs and the SI was greater than the 10×EC_50_ threshold that was targeted. The general SAR trends around this series for activity against *L*. *major* are summarized in **Fig 6**.

### Trypanosoma brucei brucei

**Fig 7:**
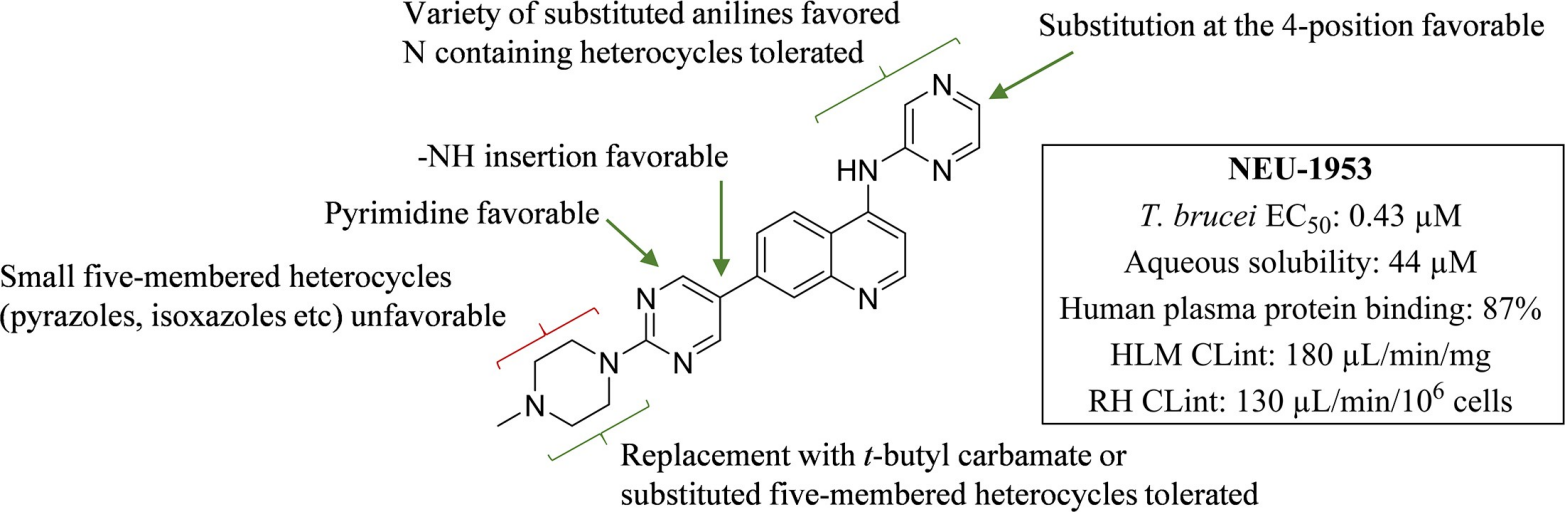
SAR summary around this series for potent anti *T*.*b*. *brucei* activity.

With a reduction of the lipophilicity of the head group several sub-micromolar inhibitors of *T*.*b*. *brucei*, including *para* substituted pyridines (**5**, **11** and **12**; EC_50_ values ranging from 0.27–0.70 μM), pyrazines (**3** and **4**; EC_50_: 0.62 and 0.91 μM respectively) and several substituted anilines (**6**, **8** and **10**; EC_50_ values ranging from 0.22–0.65 μM) were identified (**Fig 2**). Replacement of the phenylsulfonamide with the pyrimidine (**14**; EC_50_: 0.0060 μM) (**Fig 3**) led to a 10-fold improvement in potency over **1** (EC_50_: 0.079 μM). Additionally, incorporation of an -NH linker led to compounds with an improvement in potency (**17**–**20**; EC_50_ values ranging from 0.098–0.16 μM). A combination of the pyrazine head group with the pyrimidine tail (**22**; EC_50_: 0.43 μM) (**Fig 4**) produced a sub-micromolar inhibitor of *T*.*b*. *brucei* proliferation. This compound had a significantly improved LLE above the desired range (4.3), and improved aqueous solubility (44 μM), though HLM (Cl_int_: 180 μL/min/mg) and RH clearance (Cl_int_: 130 μL/min/10^6^ cells) are still of concern and require further optimization. The general SAR trends around this series for activity against *T*.*b*. *brucei* are summarized in **Fig 7**.

Given the activity observed against *T*.*b*. *brucei*, a selection of compounds that demonstrated sub-micromolar inhibition of *T*.*b*. *brucei* were further profiled against *T*. *congolense* and *T*. *vivax*, which are the main causative agents for African animal trypanosomiasis (AAT) (**Table 1**), to determine how well the activity translated to these closely related kinetoplastids [5]. All of the analogs screened were observed to be sub-micromolar against both *T*. *congolense* and *T*. *vivax*. Compounds **22** and **23** were observed to be more potent than diminazene for *T*. *congolense* (EC_50_: 0.050 μM), and *T*. *vivax* (EC_50_: 0.040 μM), respectively. None of the compounds tested were more potent than isometamidium against either species.

**Table 1:** Biological activity against *T*. *congolense* and *T*. *vivax*.

ID	*T*. *b*. *brucei* EC_50_ (μM)	*T*. *congolense* EC_50_ (μM)	*T*. *vivax* EC_50_ (μM)
**1**	0.080	0.14	0.040
**6**	0.22	0.86	0.16
**14**	0.0060	0.16	0.20
**15**	0.14	0.39	0.21
**16**	0.11	0.47	0.15
**21**	0.15	0.83	0.29
**22**	0.43	0.050	0.72
**23**	0.91	0.14	0.040
**Diminazene**	0.0090	0.13	0.10
**Isometamidium**	nt	0.0060	0.00012

nt = not tested

We selected the most promising *T*. *brucei* inhibitor (**14**) for pharmacokinetic (PK) analysis on the basis of its high potency (EC_50_: 0.0060 μM). PK parameters were measured for both brain and plasma following a single intraperitoneal (i.p.) dose of 10 mg/kg (see **Supplementary information, S5 and S6 Tables**). To achieve efficacy in the *in vivo* acute HAT model, we were aiming to exceed 10×EC_50_ for 6–8 h, though at this dose we were only able to exceed these levels for 4 h in plasma (see **Supplementary information, S1 Fig**). Further, **14** had an excellent brain to plasma exposure ratio of 7.4, which was encouraging given CNS exposure is required to treat stage 2 HAT infections, when parasites have crossed the blood-brain barrier [16].

Given that the PK results of **14** indicated a higher dose would be required to achieve 10×EC_50_, we administered a single i.p. dose of 25 mg/kg in a murine model of acute HAT. Control mice received an i.p. dose of drug vehicle (dimethylsulfoxide, 3.4 mL/kg). There was no evidence of toxicity and a 10-fold reduction in parasitemia was observed in 75% of mice dosed with **14** while one had no detectable parasitemia (see **Supplementary information, S2 Fig**).

**Fig 8:**
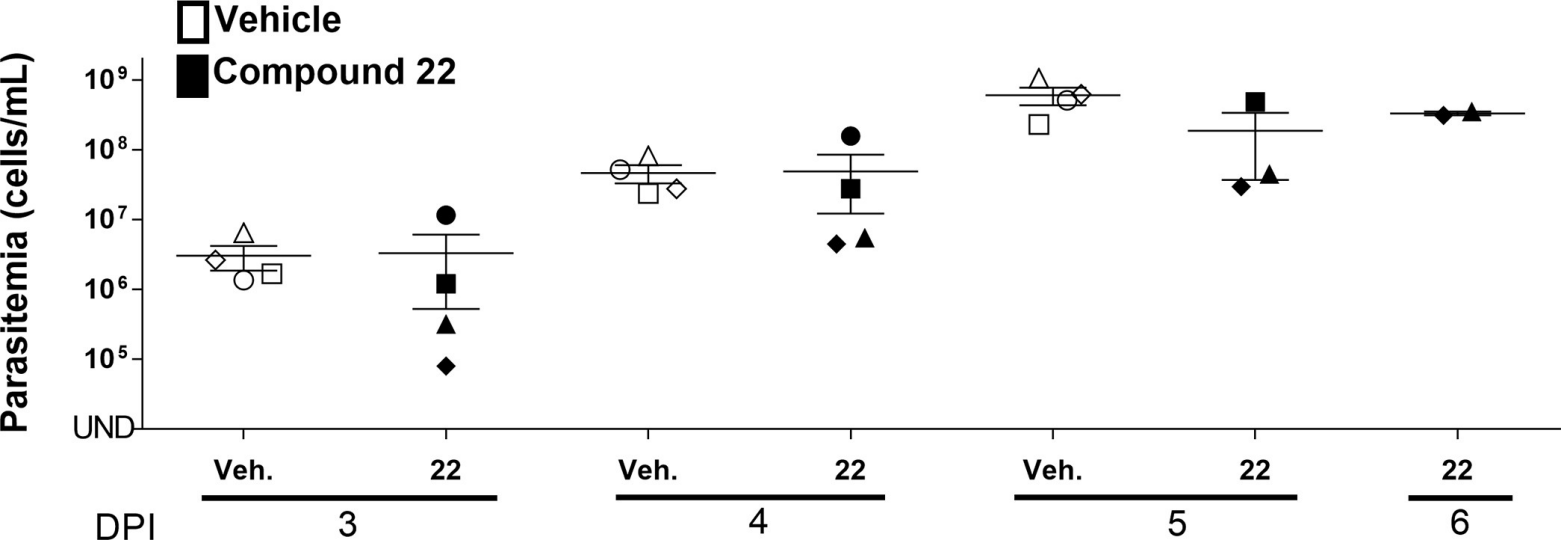
Parasitemia levels of *T*. *b*. *brucei* infected mice treated with 60 mg/kg 22 for the first three days post-infection before the dose was increased to 70 mg/kg for the remainder of the study, compared with the control group (10% NMP and 90% PEG only). Compound **22** and vehicle were administered once p.o. on Day 1 post-infection. Parasitemia in the blood collected from the tail vein was determined on Days 2–6 post-infection. UND: Undetectable parasitemia (<2×10^4^ cells/mL), the black horizontal line in each group indicates the median parasitemia level. The different shapes are representative of each mouse in the group. NS: Not significant. The error bar indicates the standard error of the mean (SEM). The significance of the difference in the mean parasitemia of treated and untreated groups was analyzed by Student’s T-test.

Given the improvements in the overall ADME profile of **22**, coupled with its sub-micromolar inhibition of *T*.*b*. *brucei* we sought to progress it into an efficacy study. Mice were administered orally (p.o.) once daily 60 mg/kg for the first three days post-infection. Observing no signs of toxicity, the dose was increased to 70 mg/kg for the remaining three days of treatment. Control mice received a p.o. dose of drug vehicle (10% NMP and 90% PEG, 10 mL/kg). By day 5, there was no statistically significant difference between the control group and the group treated with **22** (**Fig 8**).

In summary, by screening a series of anti-*P*. *falciparum* proliferation inhibitors against kinetoplastids, we have identified several potent compounds against *T*. *cruzi*, *L*. *major*, and *T*.*b*. *brucei*. When taken in its entirety, this set of compounds generally shows low host cell toxicity and the analogs had good to excellent selectivity for the parasite of interest. Of note was the identification of **22** which exhibited potent inhibition of *T*.*b*. *brucei*, and an improved ADME profile but, when it was progressed into an *in vivo* model of acute HAT infection, failed to affect parasitemia. We are working to ascertain the reasons for the lack of translation from *in vitro* to *in vivo* and we continue to pursue further optimization of this series as anti-trypanosomal and anti-leishmanial lead compounds, the results of which will be reported in due course.

## Materials and methods

### Trypanosoma brucei

The assay was performed following a previously reported procedure [17]. Briefly, in a 96-well plate, compounds were added in triplicates at 50 μM and in serial dilutions 1:2 in HMI-9 medium. To each well, 100 μL of 2.5×10^3^ *T*. *b*. *brucei* (strain 427, received as a gift from Dr. C. C. Wang at UCSF) in HMI-9 medium was were added and incubated at 37°C, 5% CO_2_ for 48 h. Following incubation, 20 μL of PrestoBlue were added to each well and incubated for additional 4 h. Fluorescence was read at 530 nm excitation and 590 nm emission. Suramin at 100 μM was used as positive control and reference for calculation of IC_50_.

### Ethics statement

This study was carried out in strict accordance with the USA Public Health Service Policy on Humane Care and Use of Laboratory Animals and Association for Assessment and Accreditation of Laboratory Animal Care accreditation guidelines. All mice were maintained in the University of Georgia Animal Facility under pathogen-free conditions. The protocol (AUP # A2013 06-011-A10) was approved by the University of Georgia Institutional Animal Care and Use Committee.

### Mouse infection with *T*. *b*. *brucei*

Bloodstream form (BSF) *T*. *brucei brucei* CA427 parasites were maintained at densities below 1×10^6^ cells/mL in HMI-9 media supplemented with 10% fetal bovine serum (Atlanta Biologicals), 10% SERUM PLUS (Sigma), and 1% Antibiotic-Antimycotic Solution (Corning cellgro) at 37°C, 5% CO_2_ [18]. Parasites were centrifuged at 5000 xg for 3 min at room temperature and resuspended in cold 1xPBS containing 1% glucose to yield a solution of 2.5×10^6^ cells/mL. Correct cell density following resuspension was confirmed using a Z2 Coulter Counter (Beckman). Parasite viability was observed by motility with a Neubauer Bright-line hemocytometer. Cells were kept on ice until infection.

Compound **14 –**on day 0, female Swiss-Webster mice (8–10 weeks old, 20–25 g, n = 4 per group) (Harlan) were infected i.p. with 2.5×10^5^ trypanosomes by injecting 100 μL of resuspension using 26G needles. Day 1 to 3 post-infection, mice received a single i.p. dose of either drug vehicle (dimethylsulfoxide, 3.4 mL/kg) (Fisher Scientific) or drug (**14**, 25 mg/kg). Parasitemia was determined on days 2 and 3 post-infection by collecting 3 μL of blood from the tail vein.

Compound **22 –**on day 0, female Swiss-Webster mice (8–10 weeks old, 20–25 g, n = 4 per group) were infected i.p. with 1×10^5^ trypanosomes by injecting 100 μL of resuspension using 26G needles. Day 1 to 3 post-infection, mice received a single p.o. dose of either drug vehicle (10% NMP and 90% PEG, 10 mL/kg) (Fisher Scientific) or drug (**22**, 60 mg/kg). From days 4 to 6, the dose was increased to 70 mg/kg for the mice in the treatment group. Parasitemia was determined on days 3 to 6 post-infection by collecting 3 μL of blood from the tail vein.

Blood samples were mixed with 21 μL of RBC Lysis Solution (Qiagen) and incubated at room temperature for 15–45 min prior to observing for parasites by hemocytometer. Humane euthanasia by CO_2_ overdose followed by incision to form a bilateral pneumothorax was conducted on mice at study termination. All animal experimental protocols were approved and performed in accordance with the guidelines of the Institutional Animal Care and Use Committee (IACUC) at the University of Georgia.

### Trypanosoma cruzi

In a 96-well plate, 5×10^4^ 3T3 cells in DMEM without phenol red supplemented with 2% FBS were added to each well. Cells were incubated for 2 h to allow for attachment. Compounds were added in triplicates at 50 μM and in serial dilutions 1:2. To each well, 5×10^4^ *T*. *cruzi* trypomastigotes (Tulahuen strain expressing β-galactosidase) were added and incubated at 37°C, 5% CO_2_ for 96 h. Following incubation, 50 μL of 500 μM chlorophenol Red-β-D-galactopyranoside (CPRG) in phosphate-buffered saline with 0.5% NP40 was added to each well and incubated at 37°C, 5% CO_2_ for 4 h. Absorbance was read at 590–595 nm. Amphotericin B at 4 μM was used as positive control and reference for calculation of EC_50_.

### Trypanosoma congolense

*In vitro* drug sensitivity assay for *T*. *congolense* (72 h): Compounds were tested *in vitro* for efficacy against the IL3000 *T*. *congolense* (drug sensitive) strain which was originally derived from the Trans Mara I strain, isolated from a bovine in 1966 in Kenya [19]. In brief, test compounds were prepared as 10 mg/mL DMSO stocks for each assay run, data points were averaged and EC_50_ values were determined using Softmax Pro 5.2 (Molecular Devices, Inc.). Compounds were assayed in at least three separate, independent experiments and an 11-point dilution curve was used to determine the EC_50_ values. Bloodstream form trypanosomes were supported in HMI-9 medium containing 20% bovine serum and were incubated with test compounds for 69 h at 34°C in a humidified atmosphere containing 5% CO_2_. Thereafter, 10 μL of Resazurin dye (12.5 mg in 100 mL of phosphate-buffered saline, Sigma-Aldrich, Buchs, Switzerland) was added for an additional 3 h. Plates were read using a fluorescence plate reader (Spectramax, Gemini XS, Bucher Biotec, Basel, Switzerland) using an excitation wavelength of 536 nm and an emission wavelength of 588 nm.

### Trypanosoma vivax

*Ex vivo* drug sensitivity assay for *T*. *vivax* (48 h): Compounds were tested *ex vivo* against the STIB 719 / ILRAD 560 *T*. *vivax* (drug sensitive) strain which originated from the Y486 strain, isolated from a naturally infected bovine in 1976 in Zaria, Nigeria [19]. Compounds were assayed in at least three separate, independent experiments and an 11-point dilution curve was used to determine the EC_50_ values. Bloodstream form trypanosomes were harvested from a highly parasitaemic mouse (via cardiac puncture) and were incubated with test compounds for 45 h at 37°C in a humidified atmosphere containing 5% CO_2_, supported in HMI-9 medium containing 20% bovine serum. Thereafter, Resazurin dye was added to monitor trypanosome viability as described in the preceding section.

### Leishmania major

Amastigote assays were performed using pLEXSY-hyg2-luciferase *L*. *major* transfected promastigotes and the murine RAW 264.7 macrophage cell line (ATCC TIB-71, Manassas, VA). Briefly, luciferase-expressing promastigotes of each *Leishmania* species were generated by adding a luciferase encoding region to the pLEXSY-hyg2 vector (Jena Biosciences, Germany) as described [20]. One μg of the linearized pLEXSY-hyg2-luciferase vector were electroporated (480 V, 13 Ω, and 500 μF) into 4×10^7^ *Leishmania* promastigotes. Selection for stable promastigote transfectants was carried out using hygromycin B (100 μg/mL). RAW 264.7 macrophages were maintained in growth medium (Dulbecco’s Modified Eagle’s Medium supplemented with heat-inactivated 10% FBS (Life Technologies)). Cells were harvested and resuspended in growth medium at 2.0×10^5^ cells/mL, and 1×10^4^ cells/well were dispensed (final volume 50 μL) in each well of a 384 well tissue-culture treated white plate using a EVO Freedom liquid handling system (Tecan, Durham, NC). Plates were incubated at 37°C in 5% CO_2_ for 24 h after which the culture medium was removed from each well using the EVO Freedom liquid handler. Metacyclic phase pLEXSY-hyg2-luciferase*-Leishmania* promastigotes (MOI = 1:10 *L*. *major*) were added to each assay plate well and allowed to infect the RAW 264.7 macrophages. After an overnight incubation, the growth medium was aspirated and each well was washed three times with 40 μL of fresh growth medium to remove non-internalized promastigotes. After the third wash, 69.2 μL of growth medium was added to each well. Compound dilutions (final concentration ranges 0.5–10,000 ng/mL) were generated and dispensed using the liquid handling system. Compound treated plates were incubated at 37°C in 5% CO_2_ for 96 h. After incubation, 7.5 μL of a luciferin solution (Caliper Life Science, Waltham, MA) diluted to 150 μg/mL was added to each well, and the plates were incubated for 30 min at 37°C in the dark. Data were captured using an Infinite M200 plate reader (Tecan). EC_50_s were generated for each concentration response test using GraphPad Prism software 6.0.

### Drug toxicity to 3T3 cells

In a 96-well plate, 5×10^4^ NIH/3T3 (ATCC CRL-1658) cells (purchased from ATCC) in DMEM without phenol red supplemented with 2% FBS were added to each well. Cells were incubated for 2 h to allow for attachment. Compounds were added in triplicates at 200 μM and in serial dilutions 1:2 and incubated at 37°C, 5% CO_2_ for 96 h. Ionomycin at 100 μM was used as positive control and reference for calculation of EC_50_. Following incubation, 10 μL of Alamar Blue (ThermoFisher) were added to each well and cells incubated at 37°C, 5% CO_2_ for 4 h. Absorbance was read at 590–595 nm.

### Drug toxicity to HepG2 cells [21]

HepG2 cells were cultured in complete Minimal Essential Medium prepared by supplementing MEM with 0.19% sodium bicarbonate, 10% heat inactivated FBS, 2 mM L-glutamine, 0.1 mM MEM non-essential amino, 0.009 mg/mL insulin, 1.76 mg/mL bovine serum albumin, 20 units/mL penicillin–streptomycin, and 0.05 mg/mL gentamycin. HepG2 cells cultured in complete MEM were first washed with 1× Hank’s Balanced Salt Solution (Invitrogen #14175095), trypsinized using a 0.25% trypsin/EDTA solution, assessed for viability using trypan blue, and resuspended at 250,000 cells/mL. Using a Tecan EVO Freedom robot, 38.3 μL of cell suspension were added to each well of clear, cell culture-treated 384-well microtiter plates for a final concentration of 9570 liver cells per well, and plated cells were incubated overnight in 5% CO_2_ at 37°C. Drug plates were prepared with the Tecan EVO Freedom using sterile 96 well plates containing twelve duplicate 1.6-fold serial dilutions of each test compound suspended in DMSO. 4.25 μL of diluted test compound was then added to the 38.3 μL of media in each well providing a 10%-fold final dilution of compound. Compounds were tested from a range of 57 ng/mL to 10,000 ng/mL for all assays. Mefloquine was used as a plate control for all assays with a concentration ranging from 113 ng/mL to 20,000 ng/mL. After a 48 h incubation period, 8 μL of a 1.5 mg/mL solution of MTT diluted in complete MEM media was added to each well. All plates were subsequently incubated in the dark for 1 h at room temperature. After incubation, the media and drugs in each well was removed by shaking the plate over sink, and the plates were left to dry in a fume hood for 15 mins. Next, 30 μL of isopropanol acidified by addition of HCl at a final concentration of 0.36% was added to dissolve the formazan dye crystals created by reduction of MTT. Plates are put on a 3-D rotator for 15–30 mins. Absorbance was determined in all wells using a Tecan iControl 1.6 Infinite plate reader. The 50% toxic concentrations (TC_50_) were then generated for each toxicity dose response test using GraphPad Prism (GraphPad Software Inc., San Diego, CA) using the nonlinear regression (sigmoidal dose-response/variable slope) equation.

## Supporting information

S1 Table Screening data for remaining compounds in the data set. (PDF)

S2 Table Targeted properties for *T*. *cruzi* and *Leishmania* spp as set by GHIT, and our established criteria for *T*.*b*. *brucei*. (PDF)

S3 Table Lipophilic ligand efficiency (LLE) values for all compounds. (PDF)

S4 Table ADME data of all compounds. (PDF)

S5 Table Pharmacokinetic parameters of NEU-1060 (compound 14) in plasma and brain following a single intraperitoneal administration in female BALB/c mice (Dose: 10 mg/kg). (PDF)

S6 Table Brain to plasma exposure ratio of NEU-1060 (compound 14) after a single intraperitoneal administration in female BALB/c mice (Dose: 10 mg/kg). (PDF)

S1 Fig Mean plasma and brain concentration-time profiles of NEU-1060 (compound 14) following a single intraperitoneal administration in female BALB/c mice (Dose: 10 mg/kg). (PDF)

S2 Fig Parasitemia levels of *T*. *b*. *brucei* infected mice treated with 25 mg/kg NEU-1060 (compound 14), compared with the control group (dimethylsulfoxide (DMSO) only). (PDF)
