# Supplementary material for: Anilinoquinoline based inhibitors of trypanosomatid proliferation
Source: PLoS Negl Trop Dis. 2018 Nov 26;12(11):e0006834. doi: 10.1371/journal.pntd.0006834 (PMC6283615; doi:10.1371/journal.pntd.0006834)
Supplement: S1 Table — (PDF) [file pntd.0006834.s001.pdf]

Table S1. Screening data for remaining compounds in the data set

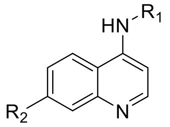

Molecule Name	R₁ (head)	R₂ (tail)	*L. major* amastigote EC_{50} (μM)[a]	*T.b. brucei* EC_{50} (μM)[b]	*T. cruzi* amastigote EC_{50} (μM)[b]	3T3 TC_{50} (μM)	HepG2 TC_{50} (μM)[c]
**NEU-2151**	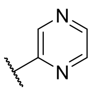	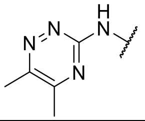	26	2.3	> 50	> 100	22
**NEU-2150**	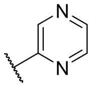	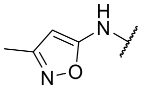	31	29	21	> 100	48
**NEU-2149**	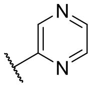	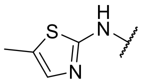	5.3	2.3	> 50	> 50	5
**NEU-2148**	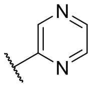	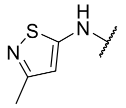	4.7	7.6	> 50	> 100	36
**NEU-2147**	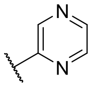	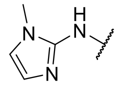	5.5	49	> 50	> 100	42
**NEU-2146**	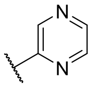	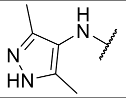	27	17	> 50	> 100	40
**NEU-2145**	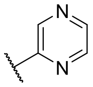	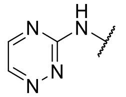	28	4.7	> 50	> 50	11
**NEU-2144**	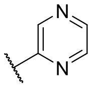	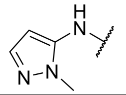	28	14	> 50	> 50	35
**NEU-2143**	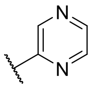	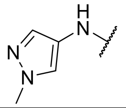	28	14	> 50	> 50	29
**NEU-2142**	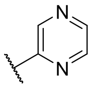	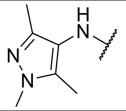	26	23	> 50	> 100	39
**NEU-2141**	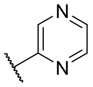	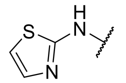	24	4.4	22	24	7.1
**NEU-2134**	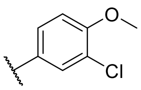	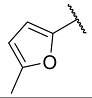	27	> 20	1.2	> 100	42
**NEU-2133**	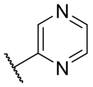	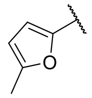	33	5.3	0.79	> 50	50

**NEU-2131**	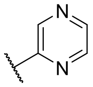	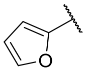	35	4.8	4.7	> 100	53
**NEU-2130**	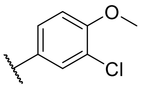	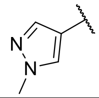	29	2.6	1.9	> 100	44
**NEU-2129**	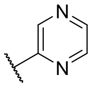	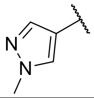	33	3.3	2.4	37	40
**NEU-2096**	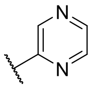	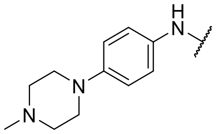	7.9	0.91	17	nd	17
**NEU-2094**	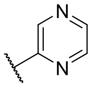	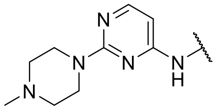	3.2	0.81	> 50	nd	21
**NEU-2093**	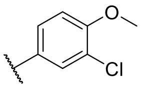	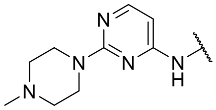	4.6	0.34	8.1	15	4.7
**NEU-2091**	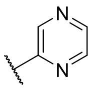	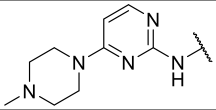	5.3	0.38	> 20	nd	22
**NEU-2090**	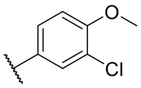	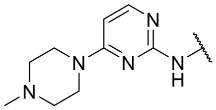	2.3	0.35	> 20	nd	7.6
**NEU-2088**	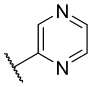	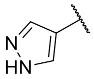	3	0.29	> 50	nd	4.5
**NEU-2087**	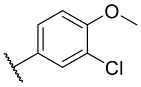	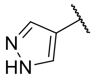	29	0.91	> 50	nd	23
**NEU-1957**	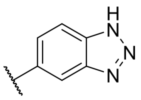	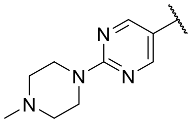	21	21	> 50	> 100	14
**NEU-1028**	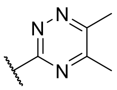	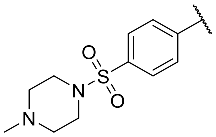	4	> 50	> 50	> 100	6.4
**NEU-1025**	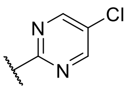	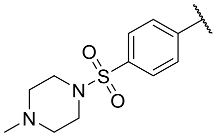	8.7	1.5	8.8	11	31
**NEU-1024**	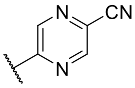	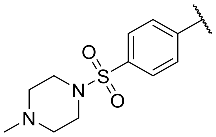	19	3	> 50	> 100	31

**NEU-1023**	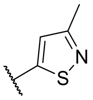	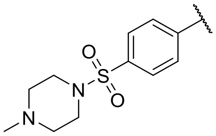	8.4	0.6	25	> 100	35
**NEU-1020**	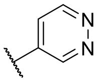	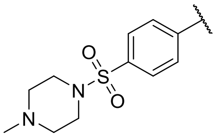	22	> 50	> 50	> 100	36
**NEU-1019**	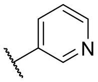	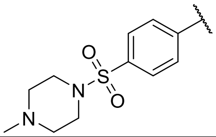	22	5.4	> 50	> 100	36
**NEU-1018**	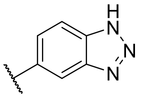	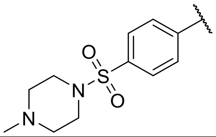	17	> 50	> 50	> 100	28

nd=not determined

[a]All r^2 values are >0.9 unless noted otherwise

[b]All SEM values within 25% except for NEU-1957 where EC_{50} *T. brucei* 21 (SEM: 8.2; 39%) and NEU-1025 where EC_{50} *T. cruzi* 8.8 (SEM: 2.6; 32%)

[c]Tested concentration ranges were determined based on compound solubility
