# Supplementary material for: Anilinoquinoline based inhibitors of trypanosomatid proliferation
Source: PLoS Negl Trop Dis. 2018 Nov 26;12(11):e0006834. doi: 10.1371/journal.pntd.0006834 (PMC6283615; doi:10.1371/journal.pntd.0006834)
Supplement: S2 Table — (PDF) [file pntd.0006834.s002.pdf]

Table S2. Targeted properties for *T. cruzi* and *Leishmania* spp as set by GHIT, and our established criteria for *T.b. brucei* [1, 2]

		T. cruzi	*Leishmania* spp.	*T. brucei*
**Potency**	EC_{50}<br>LLE (pEC_{50} – cLogP)	<10 µM	<10 µM	<1 µM<br>≥4
***In vitro* Toxicity**	HepG2 TC_{50}<br>3T3 TC_{50}	≥10 x EC_{50}<br>≥10 x EC_{50}	≥10 x EC_{50}<br>≥10 x EC_{50}	≥10 x EC_{50}<br>≥10 x EC_{50}
**ADME**	Molecular Weight (g/mol)<br>cLogP<br>LogD<br>RH Cl_{int}<br>HLM Cl_{int}<br>Aqueous Solubility (µM)<br>Plasma Protein Binding (%)	Adherence to 'rule of five' preferred	Adherence to 'rule of five' preferred	≤360<br>≤3<br>≤2<br>≤27<br>≤47<br>>10<br>≤95
**Efficacy**		>80% reduction in parasite burden after 10 doses at 50 mg/kg (PO)	>70% reduction in liver parasite burden after max 5 doses at 50 mg/kg (PO; QD, BID)	Reduction, control, or elimination of parasitemia following PO or IP dosing

References

1. Lipinski, C. A.; Lombardo, F.; Dominy, B. W.; Feeney, P. J., Experimental and computational approaches to estimate solubility and permeability in drug discovery and development settings. The article was originally published in Advanced Drug Delivery Reviews 23 (1997) 3–25.1. *Advanced Drug Delivery Reviews* **2001,** *46* (1), 3-26.
2. Katsuno, K.; Burrows, J. N.; Duncan, K.; van Huijsduijnen, R. H.; Kaneko, T.; Kita, K.; Mowbray, C. E.; Schmatz, D.; Warner, P.; Slingsby, B. T., Hit and lead criteria in drug discovery for infectious diseases of the developing world. *Nat Rev Drug Discov* **2015,** *14* (11), 751-758.
