# Supplementary material for: Anilinoquinoline based inhibitors of trypanosomatid proliferation
Source: PLoS Negl Trop Dis. 2018 Nov 26;12(11):e0006834. doi: 10.1371/journal.pntd.0006834 (PMC6283615; doi:10.1371/journal.pntd.0006834)
Supplement: S3 Table — (PDF) [file pntd.0006834.s003.pdf]

Table S3. Lipophilic ligand efficiency (LLE) values for all compounds

ID	Molecule Name	Log P	*L. major* amastigote EC_{50} (µM)	*L. major* LLE[a]	*T.b. brucei* EC_{50} (µM)	*T.b. brucei* LLE[a]	*T. cruzi* amastigote EC_{50} (µM)	*T. cruzi* LLE[a]
**1**	**NEU-961**	6.4	1.6	-0.63	0.079	0.67	0.73	-0.29
**2**	**NEU-1021**	2.7	3.1	2.8	2.5	2.9	> 50	< 1.6
**3**	**NEU-1022**	2.7	19	2.0	0.62	3.5	9.7	2.3
**4**	**NEU-1027**	2.3	20	2.4	0.91	3.8	16	2.5
**5**	**NEU-1026**	4.1	2.1	1.6	0.27	2.5	2.8	1.5
**6**	**NEU-1029**	4.6	1.4	1.3	0.22	2.1	3.8	0.86
**7**	**NEU-1030**	4.7	3.4	0.75	> 50	-0.42	> 50	< -0.42
**8**	**NEU-1031**	5.2	2.4	0.38	0.65	0.95	> 50	< -0.94
**9**	**NEU-1926**	4.0	4.4	1.4	0.52	2.3	1.9	1.80
**10**	**NEU-1927**	5.6	0.78	0.56	0.27	1.0	1.4	0.30
**11**	**NEU-1929**	3.0	3.9	2.4	0.69	3.1	9.9	2.0
**12**	**NEU-1930**	4.2	6.2	1.0	0.7	2.0	5.4	1.1
**13**	**NEU-1931**	6.4	0.2	0.27	0.48	-0.11	> 50	< -2.1
**14**	**NEU-1060**	6.2	1.5	-0.42	0.006	2.0	0.09	0.81
**15**	**NEU-1899**	6.9	0.96	-0.83	0.14	0.0039	4.7	-1.5
**16**	**NEU-1900**	5.8	0.83	0.24	0.11	1.1	0.92	0.20
**17**	**NEU-2076**	7.3	1.2	-1.4	0.12	-0.36	2.1	-1.6
**18**	**NEU-2152**	6.0	0.87	0.030	0.16	0.77	2.7	-0.46
**19**	**NEU-2086**	6.6	2.5	-1.0	0.12	0.29	2.7	-1.1
**20**	**NEU-2077**	6.6	1.2	-0.71	0.098	0.38	4.3	-1.3
**21**	**NEU-1898**	6.8	1.1	-0.84	0.15	0.024	0.6	-0.58
**22**	**NEU-1953**	2.1	2.5	3.5	0.43	4.3	6	3.1
**23**	**NEU-1956**	4.4	2	1.3	0.91	1.7	0.62	1.8
	**NEU-1018**	3.4	17	1.4	> 50	0.89	> 50	< 0.89
	**NEU-1019**	2.9	22	1.8	5.4	2.4	> 50	< 1.4
	**NEU-1020**	1.9	22	2.7	> 50	2.4	> 50	< 2.4
	**NEU-1023**	3.4	8.4	1.7	0.6	2.8	25	1.2
	**NEU-1024**	2.5	19	2.2	3	3.0	> 50	< 1.8
	**NEU-1025**	3.5	8.7	1.6	1.5	2.3	8.8	1.6
	**NEU-1028**	2.2	4	3.2	> 50	2.1	> 50	< 2.1
	**NEU-1957**	3.2	21	1.5	21	1.5	> 50	< 1.1
	**NEU-2087**	4.0	29	0.57	0.91	2.1	> 50	< 0.33
	**NEU-2088**	1.7	3	3.8	0.29	4.9	> 50	< 2.6
	**NEU-2090**	4.8	2.3	0.88	0.35	1.7	> 20	< -0.061
	**NEU-2091**	2.5	5.3	2.8	0.38	4.0	> 20	< 2.2
	**NEU-2093**	4.8	4.6	0.58	0.34	1.7	8.1	0.33

	**NEU-2094**	2.5	3.2	3.0	0.81	3.6	> 50	< 1.8
	**NEU-2096**	3.1	7.9	2.0	0.91	2.9	17	1.6
	**NEU-2129**	1.8	33	2.7	3.3	3.7	2.4	3.8
	**NEU-2130**	4.0	29	0.54	2.6	1.6	1.9	1.7
	**NEU-2131**	2.4	35	2.0	4.8	2.9	4.7	2.9
	**NEU-2133**	2.6	33	1.8	5.3	2.6	0.79	3.5
	**NEU-2134**	4.9	27	-0.35	> 20	< -0.22	1.2	1.0
	**NEU-2141**	2.5	24	2.1	4.4	2.8	22	2.1
	**NEU-2142**	1.9	26	2.7	23	2.7	> 50	< 2.4
	**NEU-2143**	1.6	28	3.0	14	3.3	> 50	< 2.7
	**NEU-2144**	1.7	28	2.9	14	3.2	> 50	< 2.6
	**NEU-2145**	1.0	28	3.6	4.7	4.4	> 50	< 3.4
	**NEU-2146**	1.8	27	2.8	17	3.0	> 50	< 2.5
	**NEU-2147**	2.0	5.5	3.3	49	2.3	> 50	< 2.3
	**NEU-2148**	2.4	4.7	2.9	7.6	2.7	> 50	< 1.9
	**NEU-2149**	3.2	5.3	2.1	2.3	2.5	> 50	< 1.1
	**NEU-2150**	1.8	31	2.7	29	2.8	21	2.9
	**NEU-2151**	1.2	26	3.4	2.3	4.4	> 50	< 3.1

[a]Lipophilic ligand efficiency (LLE): pEC_{50} - LogP
