# Supplementary material for: Anilinoquinoline based inhibitors of trypanosomatid proliferation
Source: PLoS Negl Trop Dis. 2018 Nov 26;12(11):e0006834. doi: 10.1371/journal.pntd.0006834 (PMC6283615; doi:10.1371/journal.pntd.0006834)
Supplement: S4 Table — (PDF) [file pntd.0006834.s004.pdf]

Table S4. ADME data of all compounds

ID	Molecule Name	cLogP	Polar surface area (Å²)	Aqueous Solubility (μM)	Human PPB (%)	Human Liver Microsome CLint (μL/min/mg)	Rat Hepatocyte CLint (μL/min/10^6 cells)	$LogD_{7.4}$
**1**	**NEU-961**	6.4	75	0.3	> 99	140	20	-
**2**	**NEU-1021**	2.7	100	7.3	88	-	-	3.1
**3**	**NEU-1022**	2.7	100	3.2	97	-	-	4.2
**4**	**NEU-1027**	2.3	91	4.2	94	-	-	3.2
**5**	**NEU-1026**	4.1	78	< 1	> 99	-	-	-
**6**	**NEU-1029**	4.6	75	100	99	270	44	< 1
**7**	**NEU-1030**	4.7	66	-	> 99	150	15	-
**8**	**NEU-1031**	5.2	66	-	-	290	-	-
**9**	**NEU-1926**	4	75	9	-	350	58	4.2
**10**	**NEU-1927**	5.6	75	-	> 99	280	79	4.5
**11**	**NEU-1929**	3	78	-	92	400	49	3.7
**12**	**NEU-1930**	4.2	78	-	98	390	260	4.4
**13**	**NEU-1931**	6.4	75	< 1	> 99	110	24	4.8
**14**	**NEU-1060**	6.2	66	< 1	> 99	54	24	3.4
**15**	**NEU-1899**	6.9	66	< 0.1	100	35	35	4.1
**16**	**NEU-1900**	5.8	63	12	> 99	< 3	17	4.6
**17**	**NEU-2076**	7.3	53	-	-	-	-	-
**18**	**NEU-2152**	6.0	78	-	-	-	-	-
**19**	**NEU-2086**	6.6	78	1	-	21	4.6	4.7
**20**	**NEU-2077**	6.6	78	-	-	-	-	-
**21**	**NEU-1898**	6.8	73	0.2	100	29	48	4.3
**22**	**NEU-1953**	2.1	83	44	87	180	130	3.3
**23**	**NEU-1956**	4.4	66	< 1	98	170	45	4.3
	**NEU-1018**	3.5	110	< 1	98	-	-	3.4
	**NEU-1019**	2.9	78	175	85	-	-	2
	**NEU-1020**	1.9	91	> 1000	57	-	-	-0.5
	**NEU-1023**	3.4	78	< 1	99	-	-	4.2
	**NEU-1024**	2.5	120	< 1	97	-	-	3.7
	**NEU-1025**	3.5	91	4.8	99	-	-	4.1
	**NEU-1028**	2.2	100	1.5	93	-	-	3.4
	**NEU-1957**	3.2	99	< 1	91	120	51	3.3
	**NEU-2087**	4	63	2	97	8	5	3.5
	**NEU-2088**	1.7	79	1	-	130	5	2.6

	**NEU-2090**	4.8	78	570	99	-	7.2	3.3
	**NEU-2091**	2.5	95	26	-	-	9.3	2.8
	**NEU-2093**	4.8	78	-	-	45	2.3	-
	**NEU-2094**	2.5	95	2	-	-	-	2.9
	**NEU-2096**	3.1	69	810	88	-	10	2.2
	**NEU-2129**	1.8	69	4	-	25	290	2.9
	**NEU-2130**	4	52	-	-	-	-	
	**NEU-2131**	2.4	64	6	98	160	-	3.8
	**NEU-2133**	2.6	64	4	99	190	290	4
	**NEU-2134**	4.9	47	0.1	> 99	> 300	193	4.8
	**NEU-2141**	2.5	76	4	91	68	270	3.3
	**NEU-2142**	1.9	81	570	59	13	32	2
	**NEU-2143**	1.6	81	-	63	10	66	1.9
	**NEU-2144**	1.7	81	100	69	10	16	2.4
	**NEU-2145**	1	100	3	75	42	> 300	2.1
	**NEU-2146**	1.8	91	-	68	8	120	1.8
	**NEU-2147**	2	81	160	57	12	37	1.7
	**NEU-2148**	2.4	76	2	-	28	9	3.5
	**NEU-2149**	3.2	76	1	96	54	> 300	3.7
	**NEU-2150**	1.8	89	-	-	-	-	-
	**NEU-2151**	1.2	101	-	-	-	-	-
