# Supplementary material for: Anilinoquinoline based inhibitors of trypanosomatid proliferation
Source: PLoS Negl Trop Dis. 2018 Nov 26;12(11):e0006834. doi: 10.1371/journal.pntd.0006834 (PMC6283615; doi:10.1371/journal.pntd.0006834)
Supplement: S5 Table — (PDF) [file pntd.0006834.s005.pdf]

Table S5. Pharmacokinetic parameters of NEU-1060 (compound 14) in plasma and brain following a single intraperitoneal administration in female BALB/c mice (Dose: 10 mg/kg)

Compound	Dose (mg/kg)	Route	Matrix	T_{max} (hr)	C_{max} (ng/mL)	AUC_{last} (hr*ng/mL)	AUC_{inf} (hr*ng/mL)
NEU-1060	10	i.p.	Plasma	0.25	73.98	467.90	NC
			Brain*	4.00	210.77	3474.83	NC

*The density of brain homogenate was considered as 1 which is equivalent to plasma density (1);
Brain conc. and exposure expressed as ng/g and hr.ng/g, respectively.
NC – not calculated due to improper elimination phase
