# Supplementary material for: Anilinoquinoline based inhibitors of trypanosomatid proliferation
Source: PLoS Negl Trop Dis. 2018 Nov 26;12(11):e0006834. doi: 10.1371/journal.pntd.0006834 (PMC6283615; doi:10.1371/journal.pntd.0006834)
Supplement: S6 Table — (PDF) [file pntd.0006834.s006.pdf]

Table S6. Brain to plasma exposure ratio of NEU-1060 (compound 14) after a single intraperitoneal administration in female BALB/c mice (Dose: 10 mg/kg)

Compound	Dose (mg/kg)	Route	Matrix	C_{max} (ng/mL)	C_{max} ratio	AUC_{last} (hr*ng/mL)	Brain to plasma exposure ratio
NEU-1060	10	i.p.	Plasma	73.98	2.85	467.90	7.43
			Brain*	210.77		3474.83	

*The density of brain homogenate was considered as 1 which is equivalent to plasma density (1); Brain conc. and exposure expressed as ng/g and hr.ng/g, respectively.
