# Supplementary material for: Anilinoquinoline based inhibitors of trypanosomatid proliferation
Source: PLoS Negl Trop Dis. 2018 Nov 26;12(11):e0006834. doi: 10.1371/journal.pntd.0006834 (PMC6283615; doi:10.1371/journal.pntd.0006834)

Figure S1. Mean plasma and brain concentration-time profiles of NEU-1060 (compound 14) following a single intraperitoneal administration in female BALB/c mice (Dose: 10 mg/kg)

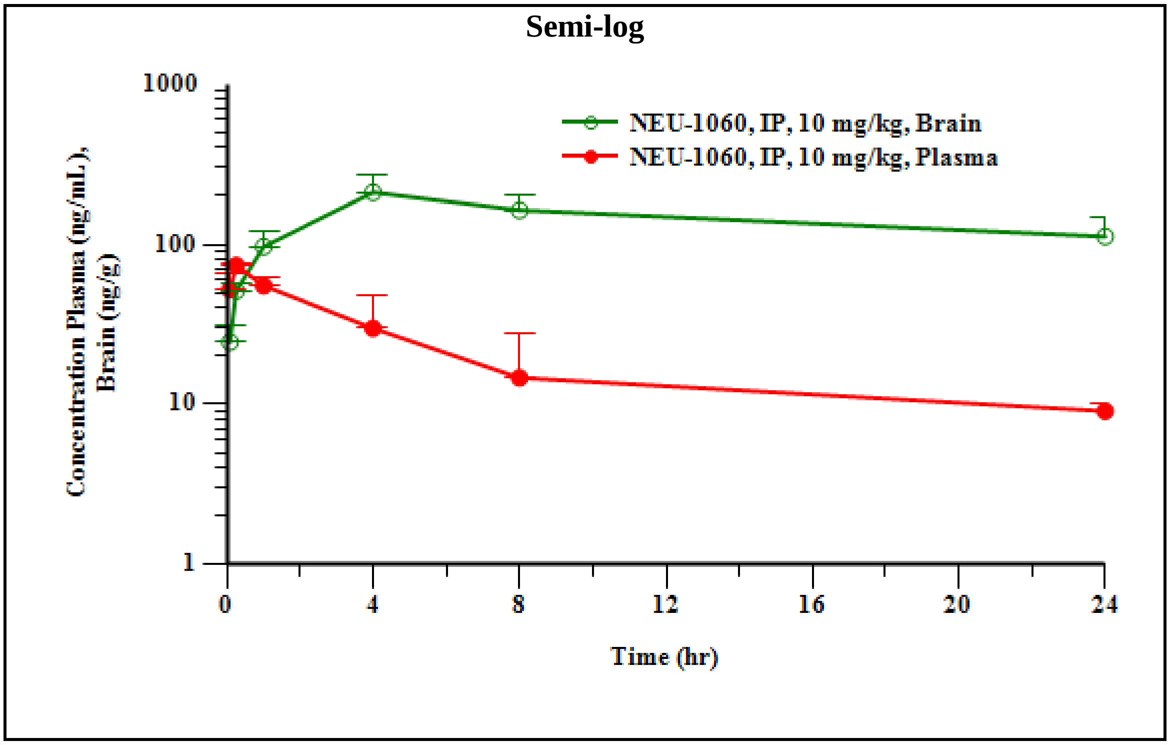

Supplement: S1 Fig — (PDF) [file pntd.0006834.s007.pdf]
