# Supplementary material for: Anilinoquinoline based inhibitors of trypanosomatid proliferation
Source: PLoS Negl Trop Dis. 2018 Nov 26;12(11):e0006834. doi: 10.1371/journal.pntd.0006834 (PMC6283615; doi:10.1371/journal.pntd.0006834)
Supplement: S2 Fig — (PDF) [file pntd.0006834.s008.pdf]

Figure S2. Parasitemia levels of *T. b. brucei* infected mice treated with 25 mg/kg NEU-1060 (compound 14), compared with the control group (dimethylsulfoxide (DMSO) only)

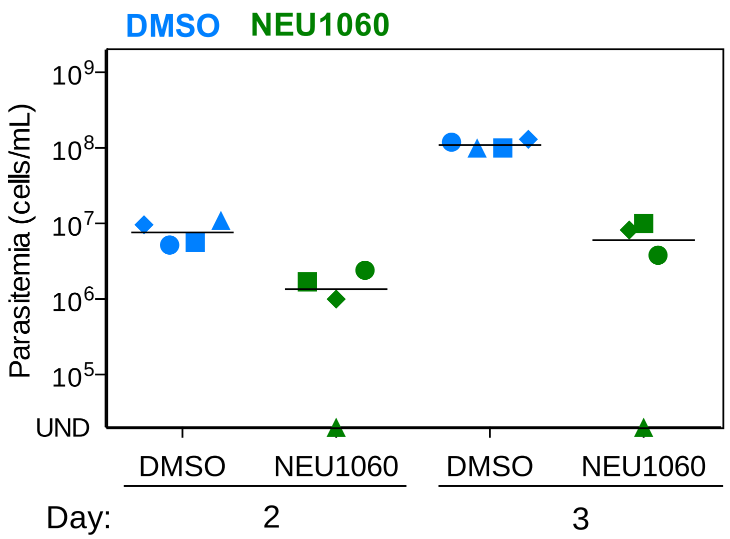

NEU-1060 (compound **14**) and DMSO were administered once i.p. on Day 1 post-infection. Parasitemia in the blood collected from the tail vein was determined on Days 2 and 3 post-infection. UND: Undetectable parasitemia (<2 x 10^4 cells/mL), the black horizontal line in each group indicates the median parasitemia level. The different shapes are representative of each mouse in the group.
